# Host Associations of *Culicoides* Biting Midges in Northeastern Kansas, USA

**DOI:** 10.3390/ani13152504

**Published:** 2023-08-03

**Authors:** Bethany L. McGregor, Aaron Lewis

**Affiliations:** 1 Arthropod-Borne Animal Diseases Research Unit, Center for Grain and Animal Health Research, USDA-ARS, Manhattan, KS 66502, USA; 2 College of Veterinary Medicine, Purdue University, West Lafayette, IN 47907, USA

**Keywords:** *Culicoides*, biting midge, vector–host interactions, ecology, blood meal analysis

## Abstract

**Simple Summary:**

*Culicoides* biting midges are small biting flies that can spread diseases to livestock. Understanding which animal species midges feed on in nature is important for assessing the risk of disease spread. In this study, we used molecular methods to determine host associations for five midge species. Midges were found to feed heavily on either birds or mammals, with a small degree of crossover for all species. The size of the bloodmeal was found to make a bigger difference in successfully detecting the host than did the digestion stage. These results indicate that species that were found to feed heavily on birds are unlikely to be vectors of mammalian diseases and provide further evidence of the vector status of the mammalian-feeding midge species.

**Abstract:**

*Culicoides* biting midges (Diptera: Ceratopogonidae) are hematophagous flies that transmit several viruses of veterinary concern to livestock. Understanding blood feeding behaviors is integral towards identification of putative vector species and preventing the transmission of these pathogens. PCR-based blood meal analysis was conducted on 440 blood-engorged *Culicoides* midges collected in northeastern Kansas, with 316 (71.8%) returning non-human vertebrate identifications at the ≥95% identity match level. Broadly, *Culicoides sonorensis*, *Culicoides stellifer*, and *Culicoides variipennis* were found to feed heavily on mammalian hosts, while *Culicoides crepuscularis* and *Culicoides haematopotus* fed on avian hosts. The blood meals in all specimens were graded prior to DNA extraction to determine whether blood meal size or digestion status significantly impacted the likelihood of a quality host match. Size had a significant impact on the likelihood of a quality match at grades 3–5, whereas digestion only significantly impacted outcomes at the most extreme grade. These vector–host dynamics have not previously been studied in *Culicoides* collected in Kansas, which represents a unique tallgrass prairie biome within the United States that is heavily interspersed with livestock operations. Based on these data, the highly abundant species *C. crepuscularis* and *C. haematopotus* are unlikely to be major vectors of mammalian viruses.

## 1. Introduction

Biting midges in the genus *Culicoides* are small, blood-feeding Dipterans which can transmit numerous pathogens of veterinary importance. In the United States, the primary *Culicoides*-borne pathogens of interest include bluetongue virus (BTV), which primarily affects sheep and white-tailed deer; epizootic hemorrhagic disease virus (EHDV), which affects white-tailed deer, mule deer, and pronghorn antelope; and vesicular stomatitis virus (VSV), which causes disease in horses, swine, and cattle [1]. While the host range for each of these viruses varies, they all primarily infect large hooved mammals, making these hosts particularly susceptible targets for blood feeding midges during outbreak periods.

The host associations of many North American *Culicoides* species are poorly understood, although there is evidence that midges can show varying degrees of association with different host groups. Class-level associations have been documented for several midge species, although strict mammalophilic or ornithophilic habits appear to be rare, based on these investigations [2,3]. In situations with well characterized host communities, host associations at the species level have been identified, indicating that some midge species may feed more preferentially on certain hosts when they are available [4]. Host associations and feeding habits are a vital part of understanding not only the ecology of vector insects but also the potential for disease transmission within a vector population.

Vectorial capacity (VC) is a measure of the efficiency of vector-borne disease transmission that takes into account both intrinsic factors, such as vector competence and the extrinsic incubation period, as well as extrinsic factors like vector density, the daily survival rate, and the daily blood feeding rate [5]. The inclusion of each of these variables ensures that the full breadth of factors contributing to transmission is considered in determining the likelihood of disease being spread from a vector to a host. For example, one study on *Plasmodium yoelii* infection in *Anopheles stephensi* mosquitoes found that increased temperature shortened the EIP, which should favor increased transmission. However, increased temperatures also had a nonlinear impact on vector competence, which ultimately reduced the VC at high temperatures [6]. The blood feeding rate in particular is an important part of the VC equation, as this variable is squared to represent the two feedings necessary for the transmission of a pathogen. First, the acquisition of the pathogen by a vector from an infected host, and then subsequent transmission to a naïve host. The inclusion of this squared variable underscores the importance of understanding host associations and blood feeding ecology. If there is a weak association between a vector and the susceptible hosts for a pathogen, the VC is reduced and the likelihood of transmission decreases.

Historically, host associations for biting arthropods were determined through the use of various non-specific serological assays that mainly differentiated at the family or order level [7] or through field studies employing tethered or caged hosts used as lures [8]. In both methods, the information gathered is limited only to the specific hosts that are actively being studied. Molecular blood meal analysis, which generates broad vertebrate matches by targeting mitochondrial genes such as 16s, cytochrome c oxidase subunit 1 (COI), and cytochrome oxidase B (CytB), is a modern method that provides a relatively unbiased determination of host blood meal source. Molecular blood meal analysis has been used for a variety of blood-feeding Dipteran taxa, including mosquitoes, sand flies, stable flies, tsetse flies, and biting midges [9,10,11,12,13,14].

While molecular blood meal analysis provides an unbiased view of host breadth, the limitations of this method have also been investigated in some taxa. In sand flies (Diptera: Psychodidae), successful molecular identification of a host was linked to the digestion stage of the blood meal rather than the size of the blood meal present [9]. In mosquitoes, decreasing size in smaller mosquitoes was found to lead to decreased ability to identify hosts, compared with larger mosquitoes, especially when the smaller blood meal was partially digested [14]. In biting midges, time post-feed significantly impacted amplification success, although the authors found that long term storage of samples (up to 9 months) and storage temperature (ambient vs. −20 °C) did not significantly affect amplification success [15].

In the present study, molecular blood meal analysis was conducted on blood-engorged *Culicoides* biting midges collected in agricultural and sylvatic habitats in Northeastern Kansas, USA, to investigate broad host associations of particularly abundant midge species. All blood meals were characterized by blood meal size and digestion status to further investigate how these two metrics impacted blood meal amplification and successful host match.

## 2. Materials and Methods

**Figure 1:**
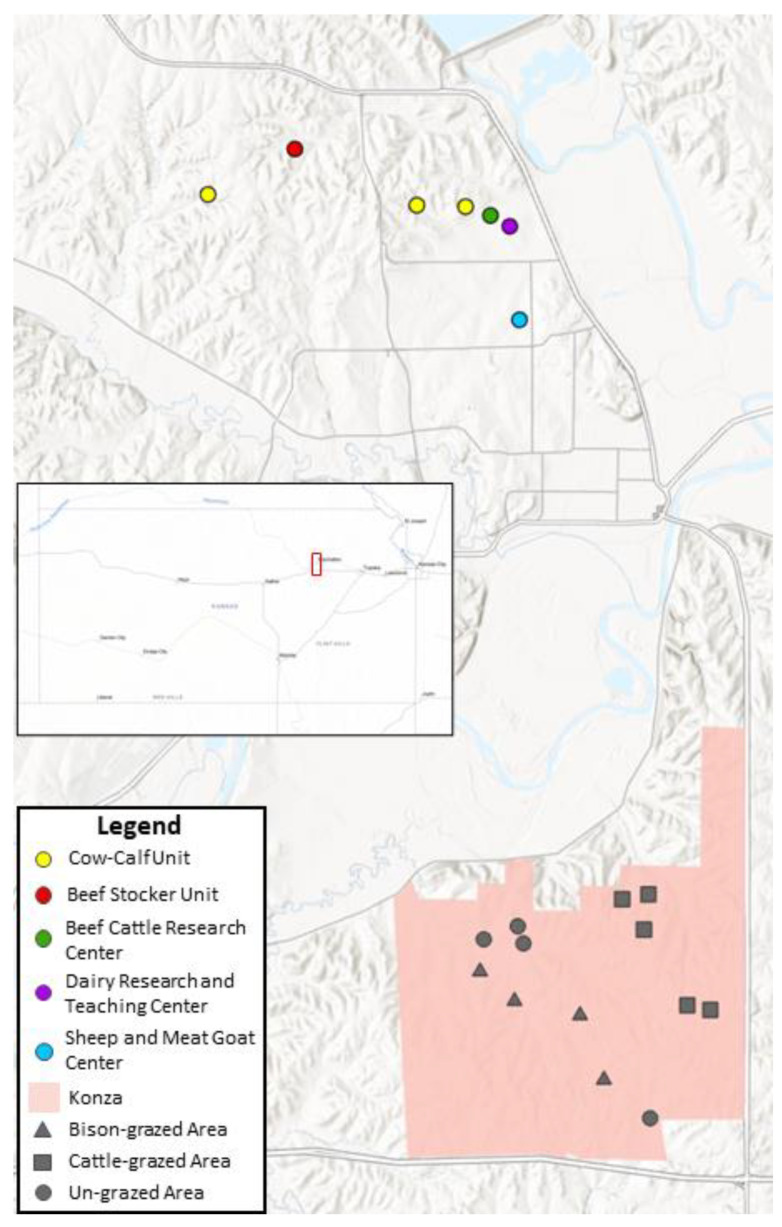
Map of collection locations, including the Konza Prairie Biological Station and the Kansas State University Animal Science and Industry facilities. Inset shows the location of the map within the state of Kansas. Map generated using ArcGIS Pro 3.1 software and included basemaps.

**Figure 2:**
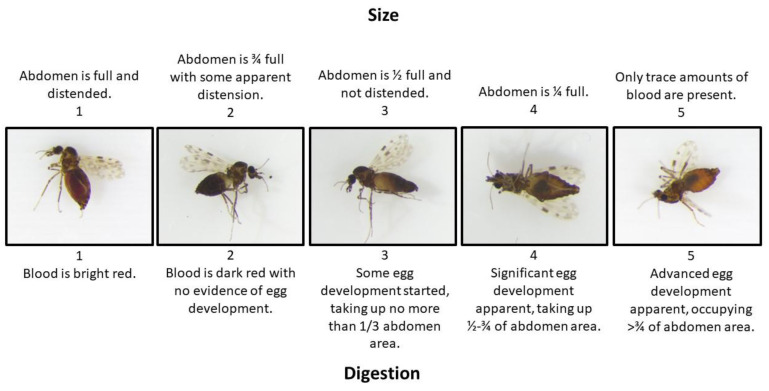
Examples of sizes and digestion grades. These examples were chosen, since their size and digestion scores matched, to make visualization easier. Most samples did not have parallel size and digestion scores.

Field collections were conducted at two sites in northeastern Kansas, USA between June 2020 and October 2021. The first site was the Konza Prairie Biological Station (KPBS), a 3487 ha expanse of native tallgrass prairie habitat that has watersheds dedicated to bison and cattle grazing as well as un-grazed areas. Twelve traps were operated at KPBS, including four in the un-grazed portion of the property, five in the cattle-grazed section, and four in the bison-grazed section. The second trapping location was the Animal Science and Industry teaching facilities at Kansas State University, located in Manhattan, KS. Ten traps were set at the KSU sites, including at the Sheep & Meat Goat Center (1), the Dairy Teaching and Research Center (4), the Beef Cattle Research Center (BCRC) (1), the Cow–Calf Unit (3), and the Beef Stocker Unit (1) (Figure 1). Midges were collected using CDC miniature light traps baited with UV LED arrays (BioQuip Inc., Rancho Dominguez, CA, USA). Traps were modified with a secondary mesh to exclude large insects and a cloth funnel to direct small insects into an attached conical tube containing 90% ethanol. Midges were sorted from bycatch using a stereomicroscope and identified to the species level using morphological characteristics [16,17,18]. Blood meals were then scored based on the level of digestion and the size of the blood meal (Figure 2). To control for observer bias, the same person scored all specimens. Following scoring, blood-engorged specimens were air-dried, transferred into individual 1.5 mL microcentrifuge tubes, and placed at −80 °C until further processing.

Blood-engorged midges were manually homogenized in 10 µL of 0.9% NaCl with a pestle using a pestle motor (Kimble, 749540-0000, DWK Life Sciences, Millville, NJ, USA). DNA was extracted using a Qiagen DNeasy Blood & Tissue Kit (Qiagen, Hilden, Germany) following the included spin-column protocol. The first step of the standard protocol was modified by combining the homogenate with 20 µL of proteinase K and 200 µL of PBS. The remaining steps of the protocol were followed as written. Eluted DNA was stored in a −20 °C freezer.

Host DNA was amplified by PCR using two different primer sets. Samples were initially run with primers targeting cytochrome B (F: GGACAAATATCATTCTGAGG, R: GGGTGGAATGGGATTTTGTC) [13]. Any samples that failed to amplify using the cytochrome B primer set were subsequently run using primers targeting the mitochondrial 16s rRNA gene fragment (F: GCCTGTTTACCAAAAACATCAC, R: CTCCATAGGGTCTTCTCGTCTT) [13]. These primer sets were selected due to their successful use with blood meal analysis in previous studies. All PCR products were run on a 1% agarose gel with CyberSafe stain at 110 V for 20 min and visualized under blue light for the presence of bands. Samples that produced bands were sent for commercial Sanger sequencing (Eurofins Genomics, Louisville, KY, USA). All PCR and electrophoresis assays were run with positive controls derived from horse blood samples and negative controls of molecular grade water. All chromatograms were checked for the presence of multiple peaks that would indicate the possibility of multiple blood meals, but none were detected.

Sequences were run against the GenBank database using BLASTn (NCBI, National Library of Medicine, Bethesda, MD, USA) to determine host matches for each individual blood meal. Matches were considered good quality at ≥95% identity match and ≥75% query coverage. Matches to human hosts were removed from this analysis to rule out any potential contamination during processing. The influence of digestion and size of the blood meal on successful host identification was evaluated using logistic regression, in which the size and digestion score were assigned as factors and the outcomes were either a successful match following the criteria above or an unsuccessful match/no amplification. Blood meal size and degradation were evaluated independently using R Studio version 3.5.0 (R Core Team, Vienna, Austria, 2018).

## 3. Results

A total of 440 blood-engorged *Culicoides* were collected and processed for blood meal analysis during the study period. The species for which blood meals were evaluated included *C. arboricola* Root and Hoffman (*n* = 2), *C. bergi* Cochrane (*n* = 1), *C. crepuscularis* Malloch (*n* = 45), *C. haematopotus* Malloch (*n* = 71), *C. hinmani* Khalaf (*n* = 2), *C. nanus* Root and Hoffman (*n* = 1), *C. sonorensis* Wirth and Jones (*n* = 72), *C. stellifer* Coquillett (*n* = 98), and *C. variipennis* Coquillett (*n* = 148). Out of 440 blood meals, 316 (71.8%) returned good quality, non-human matches for downstream analysis, and are described below.

**Figure 3:**
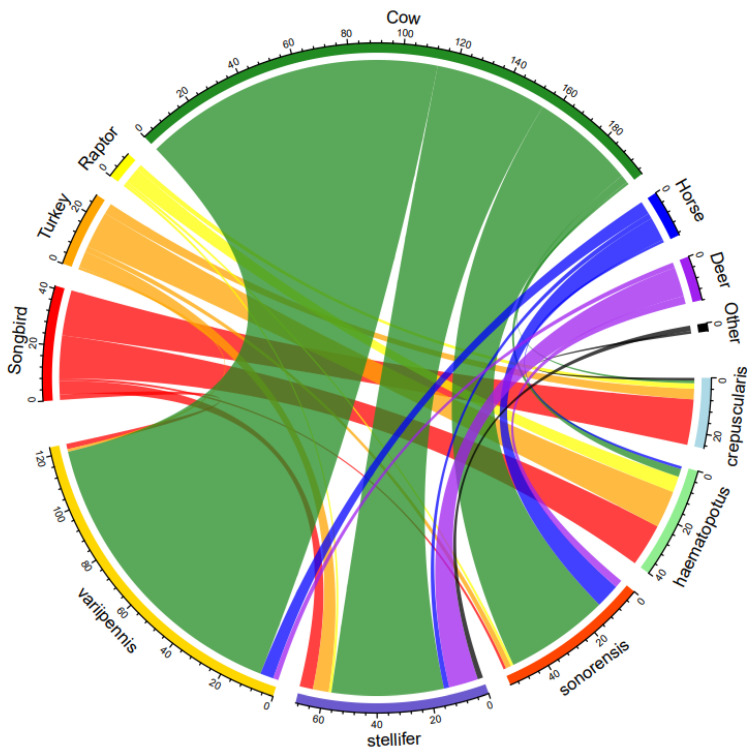
Chord diagram depicting the association of each *Culicoides* species (at the bottom) with its hosts (on the top). The size of the chord corresponds to the proportional amount of feeding on each host.

Overall, blood meals by each species were mainly split by vertebrate class (Figure 3). Species feeding primarily on mammals included *C. sonorensis* (92.7%), *C. stellifer* (82.6%), and *C. variipennis* (97.6%), while *C. crepuscularis* (92%) and *C. haematopotus* (90%) mainly fed on avian hosts. While cows were the dominant species fed on by mammalian feeders, blood meals were also taken from horses, white-tailed deer, dogs, sheep, and elk. Wild turkeys constituted the largest single-species proportion of avian bloodmeals, although blood meals were also taken from numerous wild songbird species (Table 1). *Culicoides arboricola* (*n* = 2, Wild Turkey), *C. bergi* (*n* = 1, Northern Cardinal), and *C. nanus* (*n* = 1, Summer Tanager) fed on avians; however, there were too few blood meals to draw conclusions on the broad host associations of these species. Neither of the *C. hinmani* bloodmeals met the criteria for inclusion in the dataset.

The majority of blood-engorged midges collected for this study were found at the Beef Cattle Research Center, where the most common blood-engorged species collected were *C. variipennis* (*n* = 101) and *C. sonorensis* (*n* = 41). These species were not collected in this great of an abundance at any other site in the study. Despite their association with the Beef Cattle Research Center, very few *C. sonorensis* and *C. variipennis* blood feds were collected on other cattle-associated KSU units, and none were collected within the cattle units at Konza. *Culicoides stellifer* was the only species collected from all three grazing regimens present at Konza, and it was also collected in great abundance at the KSU Cow–Calf Unit and Beef Stocker Unit. *Culicoides haematopotus* and *C. crepuscularis* were most abundant at the KSU Beef Stocker Unit and Cow–Calf Unit, respectively (Table 2).

**Figure 4:**
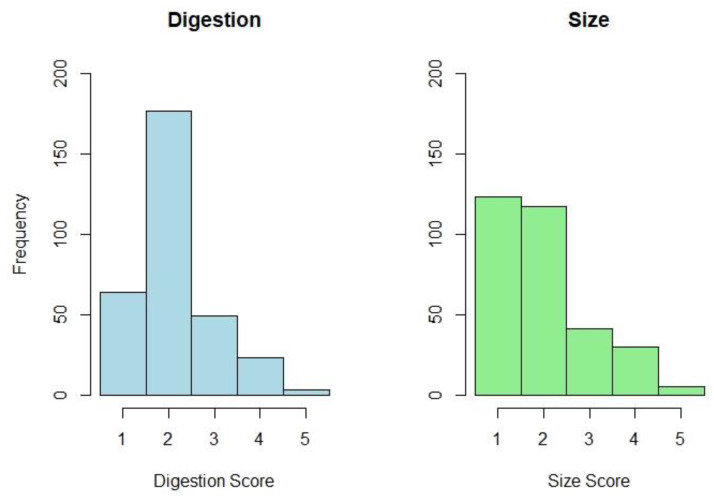
Histogram depicting the frequency of the five size and digestion scores evaluated in the dataset.

The most common grade for blood meal digestion was a 2, with the most common size grade being a 1 (Figure 4). Digestion status was only found to significantly impact host identification at the most-digested grade (5, *p* < 0.001). Size significantly impacted identification outcomes at grades 3–5 (*p* = 0.006 at grade 3, *p* < 0.001 at grades 4–5). 

## 4. Discussion

Blood meal analysis provides an unbiased opportunity to investigate the host associations of blood-feeding arthropods. In this study, the host associations of five *Culicoides* species were determined. Species such as *C. sonorensis*, *C. stellifer*, and *C. variipennis* were found to feed heavily on mammalian hosts, especially cattle. This is in stark contrast to *C. crepuscularis* and *C. haematopotus*, which were found to feed primarily on avian hosts. Although class associations were discernable, there was some degree of crossover for all species tested, with mammalian feeders taking occasional avian blood meals and avian feeders also taking mammalian meals. Additionally, blood meal results were reported for *C. arboricola*, *C. bergi*, and *C. nanus*, documenting avian feeding behavior in these species, although the sample sizes for each were too low to draw any significant conclusions for these species.

While the observed heavy cattle feeding by the mammalian feeders was not surprising considering the presence of cattle at several of the study sites, blood meal did not always correspond with the dominant host present at the sites. Despite 12 blood-engorged midges coming from the Sheep and Meat Goat Center, only one of these (*C. stellifer*) was determined to have a sheep blood meal. Four of the blood meals from this site were taken from cattle, possibly taken from an adjacent beef cattle research facility which we did not sample from for this study. Midges can disperse across several kilometers [19], and blood-engorged midges have been found to disperse hundreds of meters despite the weight added by the blood meal [20,21]. The average digestion score for the midges collected at the Sheep and Meat Goat Center was 2.3, indicating that substantial egg development had not yet begun, and oviposition was not imminent for most of these midges. These midges may have been seeking a microclimate ideal for completing digestion of the blood meal.

*Culicoides variipennis* and *C. sonorensis* were primarily found to be associated with the Beef Cattle Research Center. This facility typically houses large numbers of beef cattle in pens at a relatively high density, which is likely generating a significant amount of attractive host volatiles that these midges are following. These two species are known to emerge from the wastewater ponds present at the Dairy Teaching and Research Center located across the street (McGregor, Unpublished Data). It is surprising that blood-engorged specimens of these two species were not collected in greater abundance from the traps located at the dairy, which also houses a large concentration of cattle and is closer to the probable larval habitat from which these species are emerging. It is unclear whether this could be due to differences in handling between the beef and dairy cattle, the density of animals present, or whether there could be differences in attractiveness between cattle bred for these different purposes. 

Blood meal size was found to have a greater impact on the ability to identify blood meal source than did digestion status. This is in contrast to a study conducted on blood-engorged sand flies, which found that digestion was more important than blood meal size [9]. While the slightly smaller size of biting midges compared to sand flies could explain this difference, both groups are extremely small flies that take small blood meals, indicating that invertebrate size alone may not be resulting in this discrepancy. It is possible that digestion of midge bloodmeals does not degrade host DNA as quickly or efficiently as sand fly blood meal digestion. More research is needed to understand the comparative physiology of blood meal digestion in the Nematocera. 

From a disease transmission standpoint, these data indicate that *C. crepuscularis* and *C. haematopotus* are unlikely vectors of mammalian pathogens. A previous study found BTV-positive pools of *C. crepuscularis* and *C. haematopotus* collected during a BTV outbreak in Louisiana [22]. Another study conducted in Louisiana detected EHDV in a pool of *C. crepuscularis* [23]. The positives detected in these studies were from individuals without observable blood in the gut, which would suggest that the detected viruses were present within the insects themselves. However, based on the present study, it seems unlikely that these species would have taken the two blood meals from susceptible mammalian hosts necessary to both pick up and spread these pathogens. There were no local reports of bluetongue virus or epizootic hemorrhagic disease virus transmission during the study period. There was local transmission of vesicular stomatitis virus in 2020 [24]. No effort was made to detect any mammalian viruses from the blood-engorged midges used for this study, since there is no way to determine whether the detected pathogen was actively infecting the insect or if it was just being carried in the bloodmeal. 

While *C. crepuscularis* and *C. haematopotus* are unlikely mammalian vectors, their avian feeding habits could make these species particularly strong vector candidates for avian pathogens that can be carried by midges. One study conducted in Texas detected Onchocercidae and *Haemoproteus* DNA in *C. crepuscularis* [25], but more data are needed to determine the exact role this species and *C. haematopotus* may play in the transmission of any avian parasites in North America. Avian parasites have also been detected from field-collected *Culicoides* around the world, including *Haemoproteus* and *Plasmodium* species in Germany and Spain [26,27], *Trypanosoma*, *Leucocytozoon*, and *Plasmodium* species in Thailand [28,29], and *Haemoproteus* in Japan [30], amongst others. 

*Culicoides sonorensis*, *C. variipennis*, and *C. stellifer* were all found to feed on mammalian hosts susceptible to midge-borne viruses. *Culicoides sonorensis* is a confirmed vector for all three North American *Culicoides*-borne pathogens, so this finding simply reinforces the significance of this vector within the transmission landscape. *Culicoides variipennis* and *C. stellifer* have been implicated as putative vectors for VSV and EHDV [24,31,32], and *C. stellifer* has further been implicated as a putative vector for BTV [32,33,34]. In the absence of laboratory transmission studies, field ecological data such as these can provide further support for the consideration of these species as putative vectors. However, without vector competence studies being conducted, neither species can be considered a fully confirmed vector for these pathogens.

One elk blood meal was detected from a *C. stellifer* individual collected at the western-most cow–calf site. There are small populations of elk present in eastern Kansas, but their population numbers are low compared to other farmed and sylvatic ungulates in the area. Considering the small population size, and the thought that the closest population had been further to the west near Fort Riley, this host match was surprising. This result emphasizes the utility of using blood meal analysis, not only as a method to make inferences about invertebrate ecology, but also as a tool for monitoring vertebrate populations. Other studies have evaluated the efficacy of using arthropod blood meals for conservation biology [35] and to study changes in vertebrate communities over time [36]. Blood meal analysis results can also be used to make inferences about avian breeding and migration ranges. In this study, we identified blood meals from summer tanagers (taken by *C. crepuscularis* and *C. nanus*) from both the KSU and KPBS sites. Riley County, KS, is outside of the typical northwestern boundary of the breeding range for summer tanagers, which is valuable information for monitoring this species.

Blood meal analysis has also been used to monitor how shifts in host communities are impacting disease transmission dynamics. Blood meal analysis conducted on *Culex cedecei* mosquitoes collected in the Florida Everglades and compared with historical blood meal data showed that host use patterns had shifted from non-rodent meso-mammals to rodents after the introduction of the invasive Burmese python [36]. This shift has the potential to impact transmission of Everglades virus, a member of the Venezuelan equine encephalitis virus complex [37], an observation that was made possible due to historical blood meal data collection. In addition to changes due to introduced species, the changing climate is also anticipated to cause shifts in vector presence and abundance, disease incidence, and host ranges [38,39,40]. For these reasons, having benchmark data on host use for blood-feeding arthropods is extremely valuable in determining how the disease transmission landscape is changing over time.

## 5. Conclusions

Blood meal analysis provides researchers an opportunity to better understand host associations through an unbiased lens. These host associations can in turn be used to better understand disease transmission dynamics, make inferences about the vector status of blood-feeding arthropod species, and even to learn more about the host community that is present. The data can also be used in conjunction with other relevant data to calculate disease transmission metrics such as the vectorial capacity.

In this study, blood meal analysis data collected from northeastern Kansas, USA, revealed that *C. crepuscularis* and *C. haematopotus* are primarily feeding on birds, despite historical data indicating the presence of mammalian pathogens in field-collected individuals of these species. Based on their blood-feeding habits in this study, it is unlikely that they would take the two mammalian blood meals necessary for disease transmission to occur. However, their role as potential vectors of avian pathogens is still largely unexplored. *Culicoides sonorensis*, *C. variipennis*, and *C. stellifer* were all found to feed primarily on mammalian hosts. While *C. sonorensis* is a confirmed vector of all three North American *Culicoides*-borne viruses, *C. variipennis* and *C. stellifer* remain putative vectors that have not been fully confirmed. These data contribute to our understanding of their potential role in the transmission of mammalian viruses and further reinforce the prospect that they contribute to transmission in some capacity.

## Figures and Tables

**Table 1:** Blood meal hosts of the five most abundant *Culicoides* species collected at the Konza and KSU sites.

Site	Host Class	Host	*crepus.*	*haemat.*	*sonor.*	*stell.*	*varii.*
Konza	Avian	American crow	2	0	0	0	0
		Eastern bluebird	1	0	0	0	0
		Orchard oriole	1	0	0	0	0
		Summer tanager	0	0	0	0	0
		Turkey vulture	1	0	0	0	0
		Wild turkey	0	2	0	2	0
		Yellow-billed cuckoo	0	3	0	1	0
	Mammalian	Cow	0	1	0	14	0
		White-tailed deer	0	0	0	2	1
KSU	Avian	Barred owl	0	5	1	1	0
		Blue jay	1	1	0	0	0
		Brown-headed cowbird	1	0	0	0	0
		Common grackle	1	0	0	0	0
		Cooper’s hawk	1	0	0	0	0
		Eastern bluebird	0	3	1	1	0
		Eastern meadowlark	1	0	0	0	0
		Great horned owl	0	1	0	0	0
		House finch	1	1	0	1	0
		Mourning dove	4	0	0	0	0
		Northern cardinal	0	1	0	0	0
		Orchard oriole	1	0	0	0	0
		Summer tanager	1	0	0	0	0
		Tufted titmouse	1	2	0	0	0
		Wild turkey	4	12	2	4	1
		Yellow-billed cuckoo	1	5	0	2	2
	Mammalian	Cow	1	2	39	28	113
		Dog	1	0	0	0	0
		Elk	0	0	0	1	0
		Horse	0	1	9	2	5
		Sheep	0	0	0	1	0
		White-tailed deer	0	0	3	9	1

**Table 2:** Locations and sites of the blood-engorged midges that resulted in successful matches.

Location	Site	*arbor.*	*bergi*	*crepus.*	*haemat.*	*nanus*	*sonor.*	*stell.*	*varii.*
Konza	Bison	0	0	1	5	0	0	2	1
	Cattle	0	0	0	0	0	0	14	0
	Ungrazed	0	0	4	1	1	0	3	0
KSU	Beef Stocker Unit	2	0	2	27	0	1	19	7
	Dairy	0	0	2	0	0	8	1	13
	BCRC	0	1	0	3	0	41	3	101
	Cow–Calf Unit	0	0	13	2	0	2	23	1
	Sheep/Meat Goat	0	0	3	2	0	3	4	0

## Data Availability

Data will be made available on the Ag Data Commons following publication of this research and is also available from the authors by request.
